# Validation of the Controlling Nutritional Status (CONUT) Score and the Systemic Immune-Inflammation Index (SII) for Predicting Leakage and Surgical Complications After Head and Neck Free Flap Reconstruction: A Pilot Study

**DOI:** 10.3390/medicina61122084

**Published:** 2025-11-22

**Authors:** Jun Won Lee

**Affiliations:** Department of Plastic and Reconstructive Surgery, Kangnam Sacred Heart Hospital, College of Medicine, Hallym University, Seoul 07441, Republic of Korea; ghui8954@gmail.com

**Keywords:** controlling nutritional status score, systemic immune-inflammation index, complications, free flaps, head and neck cancer

## Abstract

*Background and Objectives*: While many factors are known to influence adverse outcomes after head and neck cancer ablation with free flap reconstruction, two low-cost inflammatory–nutritional indices—the Controlling Nutritional Status (CONUT) score and the Systemic Immune-Inflammation Index (SII)—are not widely used. This pilot study evaluated their accuracy for predicting surgical complications, including flap-site leakage, and explored data-driven cutoff values. *Materials and Methods*: We retrospectively analyzed 115 consecutive patients undergoing free-flap reconstruction. Data from medical records were obtained. Preoperative CONUT and SII were computed, receiver-operating characteristic (ROC) curves derived optimal thresholds, and associations with outcomes were tested using univariable analyses and multivariable logistic regression adjusted for age, defect area, previous radiotherapy, and flap size. Classification metrics were calculated. *Results*: SII (continuous) remained independently associated with leakage (adjusted OR ≈ 22 per log-unit increase, *p* ≈ 0.001), and CONUT (continuous) with surgical complications (adjusted OR 1.39 per point, *p* = 0.009). ROC analyses showed AUCs of 0.740 (95% CI 0.622–0.858; *p* < 0.001) for log(SII) and 0.685 (0.553–0.816; *p* = 0.006) for CONUT for overall surgical complications, and 0.780 (0.686–0.874; *p* < 0.001) for log(SII) and 0.688 (0.569–0.806; *p* = 0.002) for CONUT for leakage, without significant difference in both indices. Cutoff values for both dependent variables were 2.875 (log(SII) (≈SII 750–760) and ≥4 (CONUT). *Conclusions*: Preoperative SII and CONUT demonstrated moderate predictive performance, with notably high negative predictive values. Both indices showed no significant differences in discriminating risk, and, although the result is largely exploratory, given their high negative predictive values, they may be useful for excluding anastomotic leakage and overall postoperative complications after head and neck free-flap reconstruction.

## 1. Introduction

A mainstay in head and neck reconstruction is free tissue transfer. Several factors influence surgical outcomes, including ischemia time, operative duration, chemotherapy, age, comorbidities (such as diabetes and hypertension), smoking, prior surgeries, tumor stage, and nutritional status. These variables can significantly impact recovery and complication rates [1,2,3,4,5,6,7].

Recently, indices for malnutrition have been introduced for prognosis and estimation in various medical conditions Nutritional and immune-inflammatory markers include the Controlling Nutritional Status (CONUT) score, Systemic Immune-Inflammation Index (SII), Prognostic Nutritional Index (PNI), Geriatric Nutritional Risk Index (GNRI), Nutritional Risk Index (NRI), Systemic Inflammation Response Index (SIRI), CRP-Albumin-Lymphocyte Ratio (CALLY), and Fibrinogen-to-Albumin Ratio (FAR) have also been studied [8,9,10,11,12,13]. These indices are used in combination in some literature to improve prognostic accuracy for survival and treatment response [14,15,16].

Studies in head-and-neck microsurgery have mainly documented technical and vascular risk factors [17]. Although several inflammatory–nutritional indices have been proposed for predicting surgical complications after the free flap reconstruction, contemporary evidence specific to free-flap reconstruction remains limited [18,19,20]. Specifically, there is no study analyzing the potential for predicting adverse events of free flaps using SII.

The purpose of this pilot study was to validate whether CONUT score and SII can be applied to predict complications, including leakage after free flap reconstruction in head and neck cancer patients, to estimate accuracy metrics and cut-off values, and to explore strategies for predicting surgical outcomes using these indices.

## 2. Methods

Between 2009 and 2023, 115 consecutive patients underwent microsurgical reconstructions after head and neck cancer resections at a single cancer center. This study obtained approval from the Institutional Review Board of Kangnam Sacred Heart Hospital (HKS 2024-11-005). The medical records of the patients were retrospectively analyzed. The potential risk factors were extracted from existing literature. Patient-related factors such as sex, age, body mass index, comorbidities (hypertension (HTN), diabetes mellitus (DM), tuberculosis (TBC), hepatitis (B or C), coronary artery occlusive disease (CAOD), and cerebrovascular accident (CVA)), smoking history, alcohol consumption, ASA score, surgical area, pathology, stage, previous radiotherapy, recurred cases were reviewed. Preoperative laboratory factors included hemoglobin (Hb) as well as the components of the CONUT score (serum albumin, total cholesterol, lymphocyte count) and the SII, which incorporates neutrophil, lymphocyte, and platelet counts. All labs were obtained within the standard preoperative evaluation window; if multiple results were available, we used the measurement nearest to incision time.

Surgical and perioperative factors included the type of neck dissection, type of flap used, operation time, perioperative transfusion and vasoconstrictor use. Dependent variables included surgical complications, such as leakage, hematoma, vascular compromise, flap failure, the need for secondary flap operation, and other miscellaneous complications; however, analysis was limited to overall surgical complications and leakage due to sparse data. Each patient was counted once for the “overall surgical complication” endpoint, while overlapping events were tabulated separately for descriptive purposes.

### 2.1. Study Population

Between 2009 and 2023, 115 consecutive patients underwent immediate microsurgical reconstructions after head and neck cancer ablations were included. Inclusion criteria comprised adult patients (≥18 years) with pre-operative laboratory tests obtained before surgery and complete electronic medical records available for review. Exclusion criteria were (1) secondary or salvage reconstructions following flap failure or other postoperative complications, and (2) cases missing any key laboratory variables (albumin, lymphocyte count, or platelet count). Because the study cohort was originally defined to include only immediate primary reconstructions, no secondary or salvage cases were present at baseline, and this exclusion criterion was automatically satisfied during patient selection. In addition, no patient underwent surgery without available laboratory data; therefore, no meaningful exclusions occurred, and no data imputation was required.

### 2.2. Definition of the Variables

Flap failure was defined as the complete loss of the flap, excluding cases of partial necrosis. Leakage, or surgical site disruption, was characterized by visible fluid discharge from the surgical area or reopening of the flap suture site, regardless of the cause. Surgical site infections were not included, as there were no instances of overt abscesses or major infections necessitating urgent interventions like incision and drainage. However, infections contributing to surgical site disruption or venous congestion were accounted for within those specific variables. Complications related to arterial anastomosis were excluded since no arterial insufficiency cases occurred. Miscellaneous complications encompassed issues not classified under other categories, such as scar contractures, strictures, or arthrosis.

### 2.3. Controlling Nutritional Status (CONUT) Score

CONUT is a validated screening tool that sums points from three routinely available labs—serum albumin (g/dL), total lymphocyte count (/mm^3^), and total cholesterol (mg/dL)—to reflect protein reserves, immune status, and lipid stores, respectively. Each component is scored and added (higher = worse nutrition) [21]. We used the standard scoring scheme: albumin (≥3.5 = 0; 3.0–3.49 = 2; 2.5–2.99 = 4; <2.5 = 6), lymphocytes (≥1600 = 0; 1200–1599 = 1; 800–1199 = 2; <800 = 3), and cholesterol (≥180 = 0; 140–179 = 1; 100–139 = 2; <100 = 3). Scores were computed from the preoperative draw closest to surgery. Where needed, SI units were converted to conventional units before scoring.

### 2.4. Systemic Immune-Inflammation Index (SII)

SII was calculated as platelet count × neutrophil count/lymphocyte count (all in ×10^9^/L; if sourced as/mm^3^ we converted by ×10^−3^) [22]. SII integrates thrombocytosis and neutrophilia (pro-tumor inflammation) with lymphopenia (immunosuppression) into a single metric; higher values indicate greater systemic inflammatory burden.

For secondary cutoff analyses, each index was additionally dichotomized using ROC-derived thresholds based on Youden’s J within the study cohort; these cut points were applied uniformly across outcomes.

### 2.5. Statistical Analysis

We summarized demographics and perioperative variables using mean ± SD for continuous data and counts (%) for categorical data. Group comparisons by overall surgical complications (yes/no) used independent two-sample t-tests with Levene’s test for homogeneity of variances; when cell counts were sparse (<5 expected), categorical comparisons used Fisher’s exact (or Fisher–Freeman–Halton for r × c tables) instead of Pearson’s χ^2^. Preoperative systemic immune-inflammation index (SII) was log-transformed (log(SII)); the Controlling Nutritional Status score (CONUT) was analyzed as a continuous variable.

For risk modeling, we fitted multivariable logistic regressions for two primary outcomes—anastomotic leakage and overall surgical complications—adjusting for carefully selected confounders. The confounders were selected a priori on the basis of both clinical reasoning and univariate associations, rather than automated statistical selection. The adjustment set included advanced age, large defect area, and involvement of the aerodigestive tract. Given the limited number of adverse events, the number of covariates was intentionally constrained to preserve statistical stability and minimize overfitting.

The following factors were included:

(1) Advanced age, representing decreased physiological reserve;
(2) Larger defect area, reflecting increased operative complexity;
(3) Aerodigestive tract involvement, indicating anatomically complex surgical regions that are difficult to access and monitor postoperatively, often associated with greater risk of complications.

Models were run in two ways: (i) with log(SII) and CONUT as continuous predictors, and (ii) with ROC-derived dichotomies (cutoffs of CONUT and SII). After the confirmation of the significant trend between the dependent variables and CONUT and SII, ROC analysis was performed, and following diagnostic metrics were calculated, with 95% CIs calculated using the Wilson method for binomial proportions. Youden’s Index (YI) was used for defining cut-off values for each index and dependent variable. Model robustness was examined by internal validation using 1000-bootstrap resampling. Apparent AUCs were derived from the original models, and optimism—defined as the mean difference between bootstrap training and test AUCs—was subtracted to obtain an optimism-corrected AUC. Pairwise comparisons of correlated ROC curves for log-SII and CONUT were performed using DeLong’s test. The cutoffs were then re-entered into adjusted cutoff models.

Additional inflammatory–nutritional indices were computed for exploratory comparison: the neutrophil-to-lymphocyte ratio (NLR = ANC/lymphocytes) and prognostic nutritional index (PNI = albumin × 10 + 0.005 × lymphocytes) [9,12,15,19]. ROC analyses were performed for NLR and PNI alongside SII and CONUT, and pairwise DeLong tests were also conducted.

In addition, to evaluate potential temporal bias due to changes in surgical practice over time, cases were stratified into Early (≤2016) and Late (≥2017) periods. The year 2016 was selected as the cutoff because it coincided with a marked increase in the number of reconstructions, which in turn prompted the implementation of robust standardized surgical techniques and flap monitoring protocols. These protocols have remained largely unchanged, ensuring consistency in surgical management during the Late period. ROC analyses for SII and CONUT were conducted within each period to assess whether predictive performance changed alongside these evolving practices.

Before the main analysis, cutoff values for continuous variables were determined based on mean values and clinical judgment. Through this process, continuous variables were refined into dichotomous variables. Age was classified into two groups: under 65 and 65 or older. Pathology was refined into squamous cell carcinoma and other types. For body mass index, patients were classified into two groups based on standard definitions: normal, overweight and obese. The flap type was categorized as radial forearm free flap, and the other types of flaps. The area of cancer involvement was categorized as aerodigestive tract, and other areas. Cancer stage was grouped into stages 1 and 2, and stages higher than 2. Neck dissection methods were reclassified into selective neck dissection only, including radical neck dissection, and other methods. The flap size cutoff was set at 75 cm^2^, which was the approximate midpoint of the mean values between the two groups. Similarly, cutoff values for duration of operation was set using the same principle. The statistical analysis above was performed using SPSS software (Version 28.0, IBM Corporation, Armonk, NY, USA) and R (version 4.5.1, R Foundation for Statistical Computing, Vienna, Austria).

## 3. Results

### 3.1. The Demographics of the Cohort

Across the 115 patients, most patients were male (91/115, 79.1%) and the dominant pathology was squamous cell carcinoma (SCC 108/115, 93.9%). Non-SCC histologies were uncommon. The radial forearm flap was the primary reconstructive method (98/115, 85.2%), with smaller use of anterolateral thigh (6.1%), latissimus dorsi (3.5%), combined flaps (4.3%). Primary tumor sites were led by the tongue (24.3%), hypopharynx (21.7%), and tonsil (19.1%), with the remainder distributed across the floor of mouth (7.0%), palate (4.3%), maxilla/oropharynx (each 3.5%), glottis (2.6%), and several sites at ≤1.7%. Staging skewed advanced: stage III–IV (AJCC) comprised 87/115 (75.7%) with stage I and II accounting for 8.7% and 15.7%, respectively (Table S1).

The interval between preoperative laboratory testing and surgery ranged from 1 to 23 days, with a median of 7 days and a mean of 7.6 days. The interquartile range (4–10 days) indicates that most patients underwent surgery within approximately one week following laboratory evaluation.

Among 115 patients (85 without complications; 30 with complications), most demographic and comorbidity variables were similar between groups. Age ≥ 65 years (35.7% overall) did not differ by outcome (34.1% vs. 40.0%, *p* = 0.563). Sex distribution was comparable (female 22.4% vs. 16.7%, *p* = 0.510), as were BMI categories (normal/overweight/obese: 65/19/1 vs. 24/6/0, *p* = 0.800). Smoking history (23.5% vs. 33.3%, *p* = 0.293), alcohol use (30.6% vs. 36.7%, *p* = 0.540), ASA class (1/2/3/4: 4/63/17/1 vs. 0/18/12/0, *p* = 0.116), hypertension (31.8% vs. 30.0%, *p* = 0.858), diabetes (25.9% vs. 33.3%, *p* = 0.434), prior TB (3.5% vs. 0%, *p* = 0.297), hepatitis (3.5% vs. 3.3%, *p* = 0.960), vascular disease (14.1% vs. 13.3%, *p* = 0.915), other comorbidities (24.7% vs. 20.0%, *p* = 0.601), non-SCC pathology (5.9% vs. 6.7%, *p* = 0.877), involved area (aerodigestive vs. other; 31.8% vs. 40.0%, *p* = 0.502), recurrent disease (8.2% vs. 13.3%, *p* = 0.414), preoperative RT (7.1% vs. 13.3%, *p* = 0.294), and advanced stage (>2; 74.1% vs. 80.0%, *p* = 0.519) were also not significantly different.

Notably, flap type other than radial forearm was more common in the complication group (26.7% vs. 10.6%, *p* = 0.033 *). Large flap size (>75 cm^2^) occurred more often with complications (46.7% vs. 21.2%, *p* = 0.007 *). Perioperative vasoconstrictor use was higher in the complication group (10.0% vs. 1.2%, *p* = 0.023 *). Operation time ≥12 h (43.3% vs. 41.2%, *p* = 0.837), excess crystalloid infusion ≥7 L (6.7% vs. 1.2%, *p* = 0.105), and transfusion (60.0% vs. 43.5%, *p* = 0.121) did not differ significantly (Table 1).

Group comparisons for continuous parameters (No complications n = 64; Complications n = 51) showed several meaningful differences. Operation time was longer with complications (12.29 ± 2.52 h vs. 11.25 ± 2.47 h, *p* = 0.027 *). Absolute neutrophil count (ANC) was higher in the complication group (5035 ± 1897/µL vs. 3956 ± 1618/µL, *p* = 0.001). Total cholesterol was lower with complications (152.0 ± 41.5 vs. 169.0 ± 44.7 mg/dL, *p* = 0.038). CONUT score was higher among those with complications (2.86 ± 2.22 vs. 1.94 ± 1.58, *p* = 0.014). log-SII was also higher with complications (2.939 ± 0.326 vs. 2.750 ± 0.291, *p* = 0.001). In contrast, age (62.8 ± 8.8 vs. 60.3 ± 9.7 years, *p* = 0.078), platelets (253,667 ± 92,516 vs. 232,703 ± 64,420/µL, *p* = 0.156), lymphocytes (1446 ± 634 vs. 1607 ± 639/µL, *p* = 0.181), and albumin (4.00 ± 0.52 vs. 4.13 ± 0.46 g/dL, *p* = 0.163) were not significantly different (Table 2 and Figure S1).

A total of 30 surgical complications occurred among 115 patients (26.1%). Table S2 summarizes their distribution. Leakage/fistula accounted for the majority (66.7%), followed by total flap loss (20.0%) and minor revisionary or vascular events. Because several events overlapped in the same cases, subsequent analyses focused on two clinically relevant composite outcomes—overall surgical complications and leakage—to preserve statistical power and avoid redundancy.

### 3.2. Multivariate Logistic Regression Results

In multivariable models for overall surgical complications, both nutritional/inflammation indices retained independent associations. In the model including CONUT (adjusted for age ≥ 65, size > 75 cm^2^, and aerodigestive involvement), each 1-point increase in CONUT was associated with 40% higher odds of complications (OR = 1.404, 95% CI 1.103–1.785, *p* = 0.006). Age ≥ 65 (OR = 1.215, 0.481–3.067, *p* = 0.680), large flap size (OR = 2.275, 0.873–5.926, *p* = 0.092), and aerodigestive involvement (OR = 0.920, 0.350–2.418, *p* = 0.865) were not statistically significant.

In the model with log(SII), log(SII) was a strong independent predictor (OR = 24.041 per log unit, 4.267–135.460, *p* < 0.001), whereas age ≥ 65 (OR = 1.377, *p* = 0.516), large size (OR = 2.011, *p* = 0.172), and aerodigestive involvement (OR = 1.030, *p* = 0.953) were non-significant.

**Figure 1:**
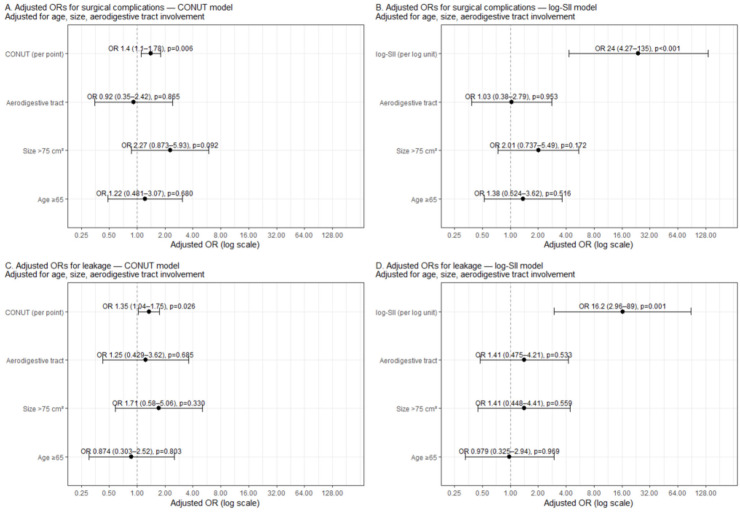
Multivariable forest plots: Adjusted odds ratios for overall complications and leakage from CONUT- and log(SII)–based models. (**A**) Overall complications—CONUT model; (**B**) Overall complications—log(SII) model; (**C**) Leakage—CONUT model; (**D**) Leakage—log-SII model. Each panel shows adjusted odds ratios (ORs) with 95% confidence intervals (CIs) on a logarithmic scale. Points indicate the adjusted OR; horizontal bars indicate the 95% CI; the vertical dashed line marks the null value (OR = 1). Models in (**A**,**C**) include age ≥ 65 years, flap size > 75 cm^2^, aerodigestive tract involvement, and CONUT (modeled per 1-point increase). Models in (**B**,**D**) include the same covariates with log-SII (modeled per 1-unit increase) instead of CONUT. Abbreviations: CONUT, Controlling Nutritional Status; SII, systemic immune-inflammation index.

For leakage, CONUT remained independently associated (OR = 1.346 per point, 1.036–1.748, *p* = 0.026), while age (OR = 0.874, *p* = 0.803), large size (OR = 1.713, *p* = 0.330), and aerodigestive tract (OR = 1.247, *p* = 0.685) were not. In the log-SII leakage model, log-SII again showed a significant association (OR = 16.239, 2.962–89.021, *p* = 0.001), with other covariates remaining non-significant (all *p* > 0.05). Collectively, these models emphasize that higher CONUT and systemic inflammation (log-SII) independently track with adverse outcomes (Table 3, Figure 1).

### 3.3. ROC-Based Performance of SII and CONUT

Discrimination for overall surgical complications (prevalence 26.1%) was moderate for both indices, with log-SII showing numerically higher AUCs than CONUT. For log-SII, the AUC was 0.780 (95% CI 0.686–0.874; *p* < 0.001), and the optimal cutoff ≥ 2.8749 (≈ SII ≥ 760) achieved sensitivity 0.733 (0.556–0.858) and specificity 0.788 (0.690–0.862), corresponding to Youden J = 0.522. The positive and negative predictive values were 0.550 (0.398–0.693) and 0.893 (0.803–0.945), respectively. For CONUT, AUC = 0.688 (95% CI 0.569–0.806; *p* = 0.002) with cutoff ≥ 4, sensitivity 0.467 (0.302–0.639), specificity 0.847 (0.756–0.908), and Youden J = 0.314 (PPV 0.519 [0.340–0.693], NPV 0.818 [0.725–0.885]).

**Figure 2:**
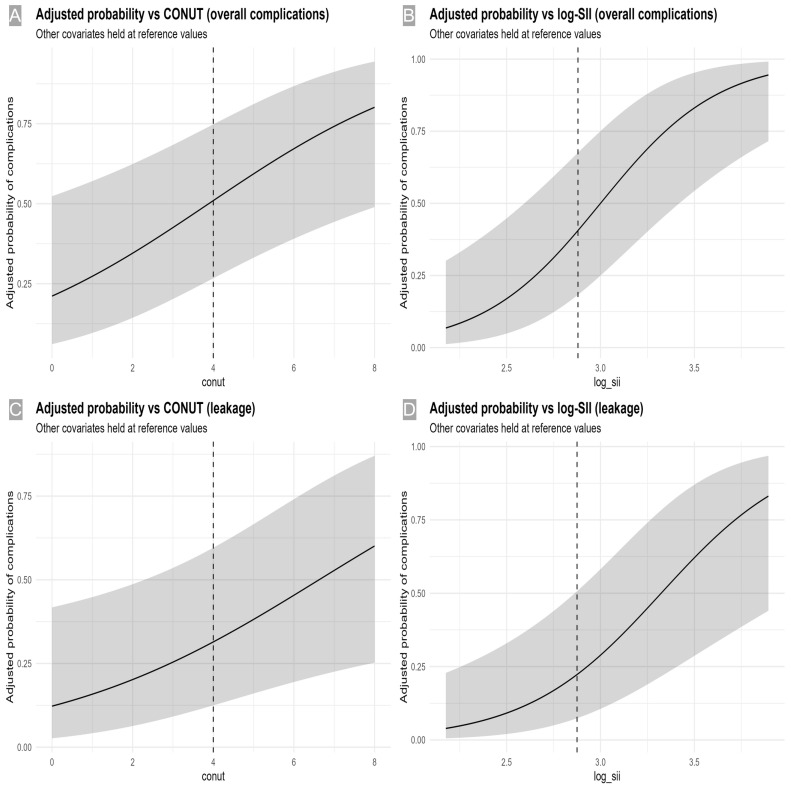
Adjusted probabilities by CONUT and log-SII. (**A**) Overall complications—CONUT: Model-predicted probability vs. CONUT (per point) with 95% CI; dashed line at cutoff 4 (other covariates at reference). (**B**) Overall complications—log-SII: Predicted probability vs. log-SII (per unit) with 95% CI; dashed line at cutoff 2.8799. (**C**) Leakage—CONUT: Predicted probability vs. CONUT (per point) with 95% CI; dashed line at cutoff 4. (**D**) Leakage—log-SII: Predicted probability vs. log-SII (per unit) with 95% CI; dashed line at cutoff 2.8749. CONUT, Controlling Nutritional Status; SII, systemic immune-inflammation index.

**Figure 3:**
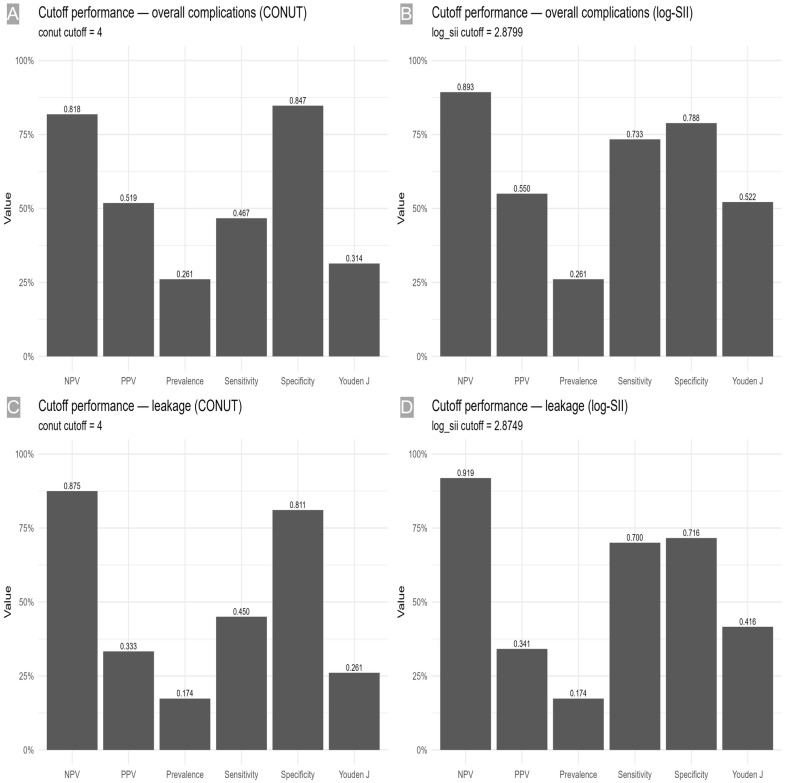
Cutoff performance at prespecified thresholds. (**A**) Overall complication—CONUT ≥ 4: Sensitivity, specificity, PPV, NPV, prevalence, and Youden’s J. (**B**) Overall complications—log-SII ≥ 2.8799: same metrics. (**C**) Leakage—CONUT ≥ 4: same metrics. (**D**) Leakage—log-SII ≥ 2.8749: same metrics. PPV, positive predictive value; NPV, negative predictive value.

**Figure 4:**
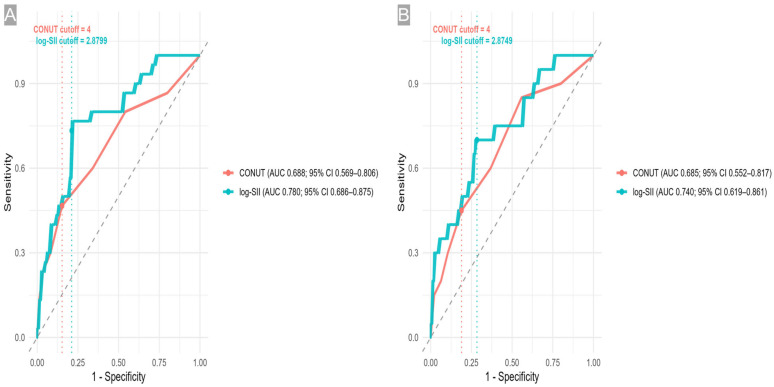
ROC comparison of log-SII vs. CONUT. (**A**) Overall complications—log-SII AUC 0.780 (95% CI 0.686–0.875); CONUT AUC 0.688 (0.569–0.806). Dotted lines: cutoffs 2.8799 (log-SII) and 4 (CONUT). (**B**) Leakage—log-SII AUC 0.740 (0.619–0.861); CONUT AUC 0.685 (0.552–0.817). Dotted lines: cutoffs 2.8749 (log-SII) and 4 (CONUT).

For leakage (prevalence 17.4%), log-SII also demonstrated acceptable discrimination (AUC 0.740 (95% CI 0.622–0.858; *p* < 0.001)). At ≥ 2.8799 (SII ≈ 750), sensitivity was 0.700 (0.481–0.855) and specificity 0.716 (0.618–0.797) (Youden J = 0.416), with PPV 0.341 (0.216–0.495) and NPV 0.919 (0.834–0.962). The CONUT index yielded AUC 0.685 (95% CI 0.553–0.816; *p* = 0.006) with cutoff ≥ 4 (sensitivity 0.450 [0.258–0.658], specificity 0.811 [0.720–0.877], Youden J = 0.261, PPV 0.333 [0.186–0.522], NPV 0.875 [0.790–0.929]) (Table 4 and Figure 2, Figure 3 and Figure 4). Internal validation demonstrated consistent discrimination after bootstrap correction (corrected AUC 0.64–0.75 for all models), confirming that the predictive models were not over-fitted (Table S3).

DeLong’s test showed no significant differences between the two indices (*p* = 0.10 and 0.54), supporting comparable discrimination (Table S4).

### 3.4. Comparison with NLR and PNI

For surgical complications, log(SII) (AUC = 0.780) and NLR (AUC = 0.775) demonstrated nearly identical discrimination (ΔAUC = +0.006, *p* = 0.81), whereas log-SII significantly exceeded PNI (AUC = 0.681, ΔAUC = +0.461, *p* < 0.001). Similarly, CONUT (AUC = 0.688) showed discrimination comparable to NLR (ΔAUC = −0.087, *p* = 0.059) but significantly higher than PNI (ΔAUC = +0.368, *p* = 0.001). For leakage, log-SII and NLR again yielded similar AUCs (0.740 vs. 0.747, ΔAUC = −0.007, *p* = 0.77), while log-SII performed significantly better than PNI (ΔAUC = +0.430, *p* < 0.001). CONUT (AUC = 0.685) displayed no difference from NLR (*p* = 0.24) but remained superior to PNI (ΔAUC = +0.375, *p* = 0.002). Overall, both log-SII and CONUT showed performance equivalent to NLR and distinctly higher than PNI (Table S5).

### 3.5. Temporal Stratification

Regarding overall surgical complications, the Early cohort (n = 26; 5/26, 19.2%) and Late cohort (n = 89; 25/89, 28.1%) showed no significant difference in crude event frequency (χ^2^ with Yates’ correction = 0.42, *p* = 0.515). Discrimination remained moderate across eras: log-SII AUC 0.848 (95% CI 0.655–1.000) and CONUT AUC 0.519 (95% CI 0.188–0.850) in Early; log-SII 0.761 (95% CI 0.649–0.872) and CONUT 0.714 (95% CI 0.590–0.839) in Late.

For leakage, the Early cohort (n = 26; 3/26, 11.5%) and Late cohort (n = 89; 17/89, 19.1%) also showed no significant difference in crude event frequency (χ^2^ with Yates’ correction = 0.36, *p* = 0.548). AUCs were stable: log-SII 0.768 (95% CI 0.450–1.000) and CONUT 0.688 (95% CI 0.318–1.000) in Early; log-SII 0.728 (95% CI 0.592–0.864) and CONUT 0.673 (95% CI 0.526–0.820) in Late (Table S6).

### 3.6. Results for Cutoff Analysis

For overall complications, the model with CONUT cutoff (adjusted for age, size, and area) showed higher odds with CONUT ≥ 4 (OR = 3.819, 1.421–10.264, *p* = 0.008), while age ≥ 65 (OR = 1.351, *p* = 0.524), large size (OR = 2.146, *p* = 0.124), and aerodigestive tract (OR = 1.159, *p* = 0.757) were not significant. In the model using SII cutoff (log(SII) ≥ 2.8799), SPSS indicator coding resulted in an OR below 1 (OR = 0.108, 0.040–0.287, *p* < 0.001). Interpreted clinically, this reflects substantially higher odds of complications in the high-SII group (inverse OR ≈ 9.26), consistent with the continuous-variable findings; age, size, and area remained non-significant.

**Figure 5:**
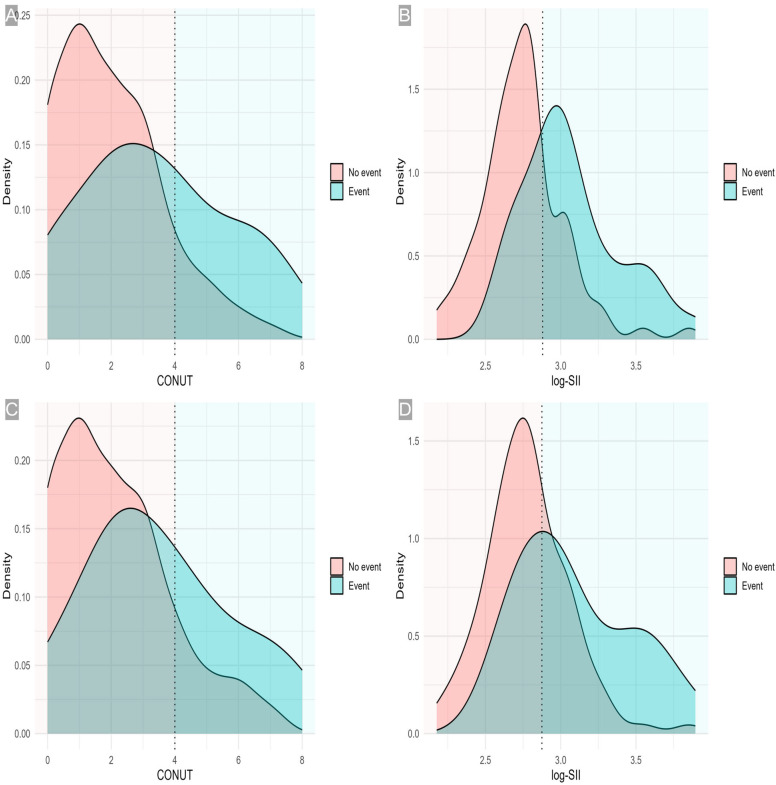

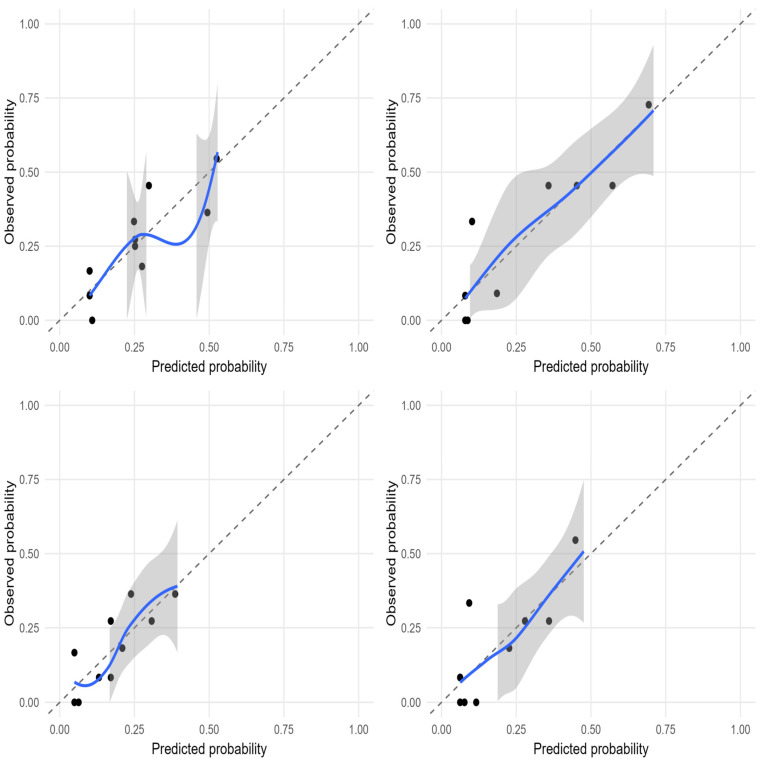
Calibration of cutoff-based multivariable models. (**A**) Overall complications—CONUT ≥ 4; (**B**) Overall complications—log-SII ≥ 2.8799; (**C**) Leakage—CONUT ≥ 4; (**D**) Leakage—log-SII ≥ 2.8749. Plots show predicted (x) vs. observed (y) probabilities from models adjusted for age ≥ 65, size > 75 cm^2^, and aerodigestive involvement; dashed line = perfect calibration; blue curve = LOESS (locally estimated scatterplot smoothing) curve; points = grouped observations; gray band = 95% CI. dashed line = perfect calibration; blue LOESS (locally estimated scatterplot smoothing) = calibration curve; points = grouped observations; gray band = 95% CI.

For leakage, CONUT ≥ 4 showed a borderline association (OR = 2.792, 0.927–8.408, *p* = 0.068), with age, size, and area not significant (all *p* > 0.30). Using the SII cutoff (log-SII ≥ 2.8749), the OR again appeared <1 due to coding (OR = 0.192, 0.065–0.566, *p* = 0.003); in clinical terms, high SII was associated with approximately 5.2-fold higher odds of leakage (inverse OR ≈ 5.21). Taken together, the cutoff analyses corroborate the continuous-scale models: elevated SII (and to a lesser degree higher CONUT) are associated with increased risk of overall complications and leakage, and the SII thresholds near ≈ 750–760 provide useful stratification in this cohort (Table 5 and Figure 5).

### 3.7. Exploratory Analysis of Specific Complications

To explore the potential clinical relevance of inflammatory–nutritional status, we examined dichotomized indices (log-SII ≥ 2.8749 ≈ SII ≥ 750; CONUT ≥ 4) across individual postoperative complications. Among patients with high SII, flap loss occurred in 14.6%, revision surgery in 34.1%, and microvascular compromise in 9.8%, compared with no such events in the lower-SII group (*p* = 0.021, 0.034, and 0.015, respectively). CONUT displayed similar directional trends but did not achieve statistical significance. Because these events were infrequent, the results should be regarded as exploratory rather than confirmatory, suggesting possible links between systemic inflammatory burden and complex postoperative courses (Table S7).

## 4. Discussion

Nutritional and inflammatory indices have been increasingly applied across a wide range of medical conditions to assess prognosis, guide treatment, and predict complications. These indices have shown clinical relevance in neurological conditions including stroke and intracerebral hemorrhage, as well as in cardiovascular diseases including heart failure, acute coronary syndrome [8,10,23]. In oncology, they have been extensively studied in patients with gastric, gastrointestinal, esophageal, colorectal, cervical, lung (NSCLC), and hepatocellular cancers, including those receiving neoadjuvant chemotherapy, radiotherapy, or curative surgery [14,15,16,22]. They are also used in evaluating outcomes in heart transplantation, Fournier’s gangrene, and IgA nephropathy [9,13]. Moreover, these markers have been explored in chronic inflammatory diseases such as rheumatoid arthritis and psoriasis, as well as in acute surgical conditions like acute cholecystitis [11,12,24]. Across these diverse conditions, nutritional and immune-inflammatory indices provide valuable insight into patient status, enabling more personalized and risk-adapted clinical care.

Recent studies have highlighted the significant impact of nutritional and inflammatory status on outcomes following free flap reconstruction in head and neck cancer. Preoperative anemia has been identified as a predictor of increased complication rates, including flap failure and extended hospital stays [25]. Nutritional markers such as hypoalbuminemia and low Prognostic Nutritional Index (PNI) are consistently associated with higher rates of postoperative complications, flap failure, and mortality [20,26]. Systemic inflammatory markers—including the neutrophil-lymphocyte ratio (NLR), systemic immune-inflammation index (SII), and systemic inflammatory marker index (SIM)—have also demonstrated prognostic value, correlating with disease-free survival and the incidence of postoperative complications [19]. Furthermore, comprehensive reviews emphasize that both undernutrition and obesity adversely affect surgical outcomes by contributing to longer operative times, impaired wound healing, and increased perioperative morbidity [4,6,27]. These findings support the integration of preoperative nutritional and inflammatory assessment into perioperative planning to optimize free flap success and overall patient recovery.

For example, Yu et al. identified low PNI as an independent predictor of flap failure in extremity reconstruction, while Luo et al. and Yen et al. confirmed its predictive value for complications in head and neck cancer surgery [26,28,29]. Although clinically useful, they typically relied on a single nutritional index, without inclusion of inflammatory or hematologic markers, and did not report receiver operating characteristic (ROC)–based cutoffs or discriminative performance metrics such as the AUC. Chargi et al. analyzed inflammatory indices in combination with radiologically quantified skeletal muscle mass, linking systemic inflammation (NLR) to sarcopenia and postoperative morbidity [30]. Rocans et al. further demonstrated that the fibrinogen-to-albumin ratio (FAR)—a coagulation-nutrition composite—was predictive of microvascular flap complications, reinforcing the interconnection between inflammatory, nutritional, and hemostatic pathways [31].

Previous literature on CONUT and SII mainly focused on survival or other microvascular complications. These two indices are not commonly used for free flap complication analysis, especially in the head and neck region. We found one analysis of CONUT in a similar cohort of 72 patients; however, it was underpowered and did not adequately control for confounding variables [18]. Furthermore, analysis for SII has not yet been conducted in the previous literature. There is a report regarding SII in the head and neck patients, but its main focus lies on the survival, not on the surgical complications [32].

The present study differs in its dual-index design and analytic rigor. By concurrently assessing CONUT (albumin, lymphocyte count, cholesterol) and SII (platelet × neutrophil/lymphocyte) within the same patient cohort, it integrates nutritional and inflammatory domains into a unified model of perioperative vulnerability. The use of ROC analysis to establish data-driven cutoff values and multivariable logistic regression adjusting for confounders such as age, flap size, defect area, and prior radiotherapy ensures a more nuanced assessment of predictive accuracy. Moreover, reporting of AUC, sensitivity, specificity, and predictive values allows for direct quantification of clinical utility—an approach not uniformly applied in earlier work. Thus, although largely exploratory, this study advances prior research by combining complementary pathophysiologic indices, applying standardized statistical evaluation. Prior studies that evaluated either nutritional or inflammatory markers in isolation may have underestimated risk in patients whose profiles were discordant (e.g., malnourished but not systemically inflamed, or vice versa). Therefore, this dual-index methodology not only advances prognostic precision but also enhances translational relevance for perioperative decision-making in head and neck free-flap reconstruction.

The current study adds to the growing body of evidence supporting the predictive value of nutritional and inflammatory indices—particularly the CONUT score and the SII—in surgical outcomes. Our study’s cutoff of CONUT ≥4 matches prior work where higher CONUT scores (≥3–5) were linked with increased morbidity and mortality [8,9,12,13,14,16,33,34]. Reported SII cutoffs in previous studies ranged from 330 to over 750, aligning with our derived threshold of SII ≈ 760 [12,15,19,22,23,24].

Using ROC-derived thresholds, CONUT ≥ 4 was associated with approximately four-fold higher odds of overall complications, highlighting the relevance of preoperative nutritional reserve. In contrast, log-SII ≥ 2.8799 (≈SII ≥ 760) identified patients with nearly nine-fold higher odds of complications, consistent with the strong association between systemic inflammation and postoperative risk. For leakage, high SII was linked to a five-fold increase in odds, while the CONUT cutoff showed only a borderline association. These results support the complementary predictive roles of SII and CONUT—reflecting inflammatory and nutritional vulnerability, respectively. However, these cutoff-based models were derived and validated within the same single-center cohort, which introduces potential overfitting and optimism bias. The apparent discriminative performance should therefore be interpreted cautiously and viewed as hypothesis-generating rather than confirmatory. Larger multicenter datasets are required to externally validate these thresholds and confirm their clinical generalizability.

To our knowledge, this study is among the first to propose specific cut-off values for CONUT and SII in the context of free flap surgical outcomes and to directly compare their predictive performance. Our findings indicate that SII may offer more reliable cut-off discrimination, as reflected by higher AUC and sensitivity. In our cohort of patients undergoing free flap reconstruction for head and neck cancer, log-transformed SII (log(SII)) demonstrated strong predictive performance for both overall surgical complications (AUC = 0.740) and leakage (AUC = 0.780), higher than CONUT (AUC = 0.685 and 0.688, respectively). Although SII yielded numerically higher AUCs than CONUT, DeLong’s tests did not detect statistically significant differences between the two indices for either endpoint, indicating comparable performance in this dataset (Supplementary Table S4). A key difference between these indices is that CONUT includes total cholesterol, which is known to be influenced by acute physiological and metabolic changes, potentially introducing variability that could affect its predictive consistency [35,36]. In contrast, SII, based solely on hematological inflammatory markers, may better capture systemic inflammation, providing a more stable prognostic indicator.

Given 30 events, inevitably the logistic models were statistically underpowered and prone to overfitting for extensive multivariable adjustment. The selection of confounders was guided by clinical experience and prior literature rather than data-driven optimization, and results should be interpreted as exploratory and internally validated rather than confirmatory. We conducted bootstrap optimism correction for adjusted models incorporating predefined clinical covariates included in the regression. Optimism-corrected AUCs remained in the moderate range, supporting the robustness of the discrimination signal while recognizing overfitting risk in small samples.

To situate SII/CONUT in the broader biomarker landscape, we added NLR and PNI as comparators (marker-only ROC analyses). SII performed similarly to NLR and generally better than PNI for both endpoints, but the DeLong pairwise comparisons should be interpreted as exploratory given the limited sample and correlated curves. Overall, these analyses suggest that host inflammatory burden and nutritional reserve—captured differently by SII and CONUT—both carry signal for perioperative risk, while emphasizing that no single index is definitively superior at this stage.

The results demonstrated particularly high negative predictive values for both CONUT and SII, indicating their effectiveness in ruling out postoperative complications. Similar high NPVs have been reported in previous studies. This is clinically valuable, as a “negative” screen (low CONUT and/or low SII) suggests patients are unlikely to develop leakage or major complications, allowing clinicians to safely de-escalate care by reducing unnecessary tests, shortening monitoring intensity, and streamlining recovery protocols. This rule-out utility is especially beneficial in busy settings with limited resources. However, the high NPV is largely influenced by the low prevalence of complications in our cohort. While this needs confirmation through external validation, given that complication rates generally remain low in similar populations, we expect NPVs to remain high across other cohorts. It is important to use CONUT and SII as adjuncts—not replacements—for clinical judgment, guiding low-risk patients to standard care and identifying high-risk individuals for targeted interventions.

A key critique concerned the predominance of radial forearm free flaps (RFFF) and potential venous/technical determinants of outcome. The RFFF emphasis was intentional to reduce anatomical and procedural heterogeneity: RFFF offers predictable vessel caliber/length, relatively uniform pedicle geometry, and short ischemia/anastomosis times—thereby minimizing flap-specific confounding when testing patient-level biomarkers. Contemporary literature underscores the effect of technical refinements regarding flap complications and failures; in our cohort, microvascular compromise was infrequent (≈2.6%), consistent with high-volume practice [17].

The extended study period (2009–2023) raises the possibility that evolving surgical pathways influenced outcomes. Complication frequencies were reported by era, and AUCs of SII/CONUT within each stratum were calculated. Despite a higher overall burden of events in the Late period, discrimination remained moderate and similar, implying that host-status indices retain predictive information across changes in perioperative practice. This mitigates concern that the observed performance is merely an artifact of early-era techniques.

Importantly, our temporal stratification (2009–2016 vs. 2017–2023) showed that SII/CONUT discrimination was maintained across eras—Early: SII AUC 0.848 (95% CI 0.655–1.000), CONUT 0.519 (0.188–0.850); Late: SII 0.761 (0.649–0.872), CONUT 0.714 (0.590–0.839)—arguing that host-status signal persisted despite procedural evolution. Collectively, these data suggest that systemic physiology (inflammation, nutrition) contributes to risk beyond purely technical variation.

The overall complication rate (26.1%) in this series was within the expected range for free-flap reconstruction in head-and-neck oncology but produced few events in each specific subtype. Because flap loss, microvascular compromise, and hematoma occurred infrequently, formal multivariable modeling was not statistically robust. Nevertheless, exploratory univariate analyses suggested that higher preoperative SII values were observed in patients who experienced revision surgery, microvascular compromise, or hematoma, whereas elevated CONUT scores showed a weaker but parallel trend. These findings, although not definitive, imply that systemic inflammatory or nutritional imbalance may predispose to vascular instability or impaired wound healing at the microanastomotic site. Given the small number of events, these associations should be interpreted cautiously and regarded as hypothesis-generating rather than confirmatory. Future multicenter datasets with larger case volumes and broader flap distributions are needed to delineate how systemic indices interact with procedure-specific risks.

There are several limitations in this study. We were unable to adjust for all potential confounders due to the limited number of surgical complication events. In the univariate analysis, flap size and vasoconstrictor use emerged as significant variables; however, vasoconstrictor use was excluded from further analysis because of the small number of events. Other confounders should be addressed in future studies. Additionally, dependent variables such as flap failure, hematoma, vascular compromise, and the need for secondary flap reconstruction could not be analyzed due to their rarity. As this is a pilot study, we plan to examine flap failure and related outcomes in an upcoming article with a larger cohort.

Beyond validation, the CONUT/SII framework supports a three-tiered risk stratification model as an adjunct decision tool: (i) rule-out (low risk, log-SII < 2.88/SII < 760 and CONUT < 4, optimized for high NPV and reduced monitoring, candidates for routine checkups), (ii) intermediate (considered for increased intensities for monitoring and drain surveillance, more frequent checkups), and (iii) rule-in (high risk, both indices elevated, requiring enhanced flap surveillance and early nutritional intervention) (Figure S2).

A prospective impact study is needed to assess whether score-guided pathways can safely reduce complications, imaging, and length of stay, while incorporating ongoing calibration and periodic model updates.

## 5. Conclusions

In this exploratory single-center study, preoperative SII and CONUT scores showed moderate ability to predict surgical complications and leakage after head and neck free flap. Both indices demonstrated high negative predictive values, supporting their potential use as adjunct tools for perioperative risk assessment. Although SII showed numerically higher AUCs, no statistically significant difference was observed between the two indices. These findings should be interpreted cautiously as preliminary evidence, warranting validation in larger, multi-institutional cohorts with diverse flap types and complication profiles.

## Figures and Tables

**Table 1:** Baseline characteristics and perioperative factors by overall surgical complications.

Variable	No Complications (n = 85)	Complications (n = 30)	Total (N = 115)	*p*-Value
Age ≥ 65	29 (34.1%)	12 (40.0%)	41 (35.7%)	0.563
Female sex	19 (22.4%)	5 (16.7%)	24 (20.9%)	0.510
BMI category (normal vs. overweight vs. obese)	65/19/1	24/6/0	89/25/1	0.800
Smoking history	20 (23.5%)	10 (33.3%)	30 (26.1%)	0.293
Alcohol use	26 (30.6%)	11 (36.7%)	37 (32.2%)	0.540
ASA class (1/2/3/4)	4/63/17/1	0/18/12/0	4/81/29/1	0.116
Hypertension	27 (31.8%)	9 (30.0%)	36 (31.3%)	0.858
Diabetes mellitus	22 (25.9%)	10 (33.3%)	32 (27.8%)	0.434
History of TB	3 (3.5%)	0 (0.0%)	3 (2.6%)	0.297
Hepatitis	3 (3.5%)	1 (3.3%)	4 (3.5%)	0.960
Vascular disease	12 (14.1%)	4 (13.3%)	16 (13.9%)	0.915
Other comorbidities	21 (24.7%)	6 (20.0%)	27 (23.5%)	0.601
Non-SCC pathology	5 (5.9%)	2 (6.7%)	7 (6.1%)	0.877
Flaps other than radial forearm flap	9 (10.6%)	8 (26.7%)	17 (14.8%)	0.033 *
Involved area (aerodigestive tract vs. other areas)	27 (31.8 *)	12 (40.0%)	39 (33.9%)	0.502
Recurrent disease	7 (8.2%)	4 (13.3%)	11 (9.6%)	0.414
Preoperative RT	6 (7.1%)	4 (13.3%)	10 (8.7%)	0.294
Advanced stage (over stage 2)	63 (74.1%)	24 (80.0%)	87 (75.7%)	0.519
Large flap size (over 75 cm^2^)	18 (21.2%)	14 (46.7%)	32 (27.8%)	0.007 *
Long operation time (12 h and above)	35 (41.2%)	13 (43.3%)	48 (41.7%)	0.837
Excess crystalloid infusion(<7 L)	1 (1.2%)	2 (6.7%)	3 (2.6%)	0.105
Perioperative transfusion	37 (43.5%)	18 (60.0%)	55 (47.8%)	0.121
Perioperative vasoconstrictor use	1 (1.2%)	3 (10.0%)	4 (3.5%)	0.023 *

* statistically significant (*p* < 0.05).

**Table 2:** Differences between groups regarding continuous variables.

Variable	Overall (n = 115)	No Complications (n = 64)	Complications (n = 51)	*p* (Two-Sided)
Age (years)	61.5 ± 9.3	60.3 ± 9.7	62.8 ± 8.8	0.078
Operation time (hours)	11.71 ± 2.54	11.25 ± 2.47	12.29 ± 2.52	0.027 *
Platelets (/µL)	242,000 ± 78,465	232,703 ± 64,420	253,667 ± 92,516	0.156
ANC (/µL)	4434 ± 1821	3956 ± 1618	5035 ± 1897	0.001 *
Cholesterol (mg/dL)	161.5 ± 44.0	169.0 ± 44.7	152.0 ± 41.5	0.038 *
Lymphocytes (/µL)	1536 ± 639	1607 ± 639	1446 ± 634	0.181
Albumin (g/dL)	4.08 ± 0.49	4.13 ± 0.46	4.00 ± 0.52	0.163
CONUT (points)	2.35 ± 1.94	1.94 ± 1.58	2.86 ± 2.22	0.014 *
log-SII	2.833 ± 0.320	2.750 ± 0.291	2.939 ± 0.326	0.001 *

* statistically significant (*p* < 0.05).

**Table 3:** Multivariate Logistic Regression Results.

Model	Predictor	*p*-Value	Exp (B)	CI 95% (Lower)	CI 95% (Upper)
Surgical complications—age, size, area, CONUT					
	Age over 65	0.680	1.215	0.481	3.067
	Size over 75	0.092	2.275	0.873	5.926
	Aerodigestive tract	0.865	0.920	0.350	2.418
	CONUT (per point)	0.006 *	1.404	1.103	1.785
Surgical complications—age, size, area, log(SSI)					
	Age over 65	0.516	1.377	0.524	3.618
	Size over 75	0.172	2.011	0.737	5.486
	Aerodigestive tract	0.953	1.030	0.380	2.793
	log-SSI (per log unit)	<0.001 *	24.041	4.267	135.460
Leakage—age, size, area, CONUT					
	Age over 65	0.803	0.874	0.303	2.523
	Size over 75	0.330	1.713	0.580	5.063
	Aerodigestive tract	0.685	1.247	0.429	3.621
	CONUT (per point)	0.026 *	1.346	1.036	1.748
Leakage—age, size, area, log(SSI)					
	Age over 65	0.969	0.979	0.325	2.944
	Size over 75	0.559	1.406	0.448	4.413
	Aerodigestive tract	0.533	1.414	0.475	4.205
	log-SSI (per log unit)	0.001 *	16.239	2.962	89.021

CI = Wald 95% confidence interval; * statistically significant (*p* < 0.05).

**Table 4:** ROC-Based Performance of log(SII) and CONUT.

**Overall Surgical Complications with Prevalence: 30/115 (26.1%)**								
Marker	AUC (95% CI)	*p*-value	Cutoff	Sensitivity (95% CI)	Specificity (95% CI)	Youden J	PPV (95% CI)	NPV (95% CI)
log(SII)	0.780 (0.686–0.874)	<0.001 *	≥2.8749 (SII ≈ 760)	0.733 (0.556–0.858)	0.788 (0.690–0.862)	0.522	0.550 (0.398–0.693)	0.893 (0.803–0.945)
CONUT	0.688 (0.569–0.806)	0.002 *	≥4	0.467 (0.302–0.639)	0.847 (0.756–0.908)	0.314	0.519 (0.340–0.693)	0.818 (0.725–0.885)
**Leakage with prevalence 20/115 (17.4%)**								
Marker	AUC (95% CI)	*p*-value	Cutoff	Sensitivity	Specificity	Youden J	PPV	NPV
log(SII)	0.740 (0.622–0.858)	<0.001 *	≥2.8799 (SII ≈ 750)	0.700 (0.481–0.855)	0.716 (0.618–0.797)	0.416	0.341 (0.216–0.495)	0.919 (0.834–0.962)
CONUT	0.685 (0.553–0.816)	0.006 *	≥4	0.450 (0.258–0.658)	0.811 (0.720–0.877)	0.261	0.333 (0.186–0.522)	0.875 (0.790–0.929)

* statistically significant (*p* < 0.05).

**Table 5:** Results for cutoff analysis.

Model	Predictor	*p*-Value	Exp (B)	CI 95% (Lower)	CI 95% (Upper)
Surgical complications—age, size, area CONUT(cutoff)					
	Age over 65	0.524	1.351	0.536	3.404
	Size over 75	0.124	2.146	0.811	5.674
	Aerodigestive tract	0.757	1.159	0.454	2.963
	CONUT 4 and over	0.008 *	3.819	1.421	10.264
Surgical complications—age, size, area, SII(cutoff)					
	Age over 65	0.623	1.285	0.472	3.497
	Size over 75	0.075	2.513	0.911	6.933
	Aerodigestive tract	0.736	1.191	0.431	3.288
	log(SSI) 2.8799 and over	<0.001 *	0.108	0.040	0.287
Leakage—age, size, area CONUT(cutoff)					
	Age over 65	0.928	0.953	0.333	2.723
	Size over 75	0.340	1.710	0.568	5.146
	Aerodigestive tract	0.405	1.548	0.553	4.334
	CONUT 4 and over	0.068	2.792	0.927	8.408
Leakage—age, size, area, SII(cutoff)					
	Age over 65	0.750	0.838	0.283	2.480
	Size over 75	0.319	1.731	0.588	5.098
	Aerodigestive tract	0.422	1.546	0.534	4.471
	log(SII) 2.8749 and over	0.003 *	0.192	0.065	0.566

CI = Wald 95% confidence interval; * statistically significant (*p* < 0.05).

## Data Availability

De-identified data can be made available from the corresponding author upon reasonable request.

## Supplementary Materials

The following supporting information can be downloaded at: https://www.mdpi.com/article/10.3390/medicina61122084/s1, Table S1. Baseline Categorical Characteristics (N = 115). Table S2. Distributions of complications. Table S3. Internal validation of adjusted models and comparison with the main result(B = 1000). Table S4. DeLong test result for comparison of performance between log(SII) and CONUT. Table S5. DeLong comparisons of log(SII) vs. NLR/PNI. Positive Δ(AUC) favors the first-listed index (log(SII) or CONUT). * Significant difference (*p* < 0.05). Table S6. Temporal stratification by era. Table S7. Exploratory binary associations between high inflammatory–nutritional indices and specific postoperative complications. Figure S1. Distributions of CONUT and log(SII) by outcome groups (violin + box). Each panel shows kernel-density violins overlaid with a boxplot (box = IQR; whiskers = 1.5 × IQR) and a black dot marking the median. *p*-values are Wilcoxon rank-sum tests (two-sided). Colors: pink = No, teal = Yes. Group sizes: No = 85, Yes = 30 (both outcomes; N = 115). (A) CONUT vs. overall complications—higher CONUT in the complication group (*p* = 0.002). (B) log-SII vs. overall complications—higher log-SII in the complication group (*p* < 0.001). (C) CONUT vs. leakage—higher CONUT in the leakage group (*p* = 0.009). (D) log-SII vs. leakage—higher log-SII in the leakage group (*p* < 0.001). Figure S2. Suggestion of potential risk-stratified monitoring and intervention pathway after free flap reconstruction based on SII and CONUT.
