# Supplementary material for: Validation of the Controlling Nutritional Status (CONUT) Score and the Systemic Immune-Inflammation Index (SII) for Predicting Leakage and Surgical Complications After Head and Neck Free Flap Reconstruction: A Pilot Study
Source: Medicina (Kaunas). 2025 Nov 22;61(12):2084. doi: 10.3390/medicina61122084 (PMC12734734; doi:10.3390/medicina61122084)
Supplement: Supplementary file 1 [file medicina-61-02084-s001.zip › final medicina-3953298-supplementary/supplementary_tables.docx]

**Table S1.** Baseline Categorical Characteristics (N=115).

| **Variable** | **Category** | **N** | **%** |
| --- | --- | --- | --- |
| **Sex** | Female | 24 | 20.9 |
|  | Male | 91 | 79.1 |
| **Pathology** | SCC | 108 | 93.9 |
|  | Adenocarcinoma | 1 | 0.9 |
|  | Adenocystic | 3 | 2.6 |
|  | Melanoma | 1 | 0.9 |
|  | Mucoepidermoid | 1 | 0.9 |
|  | Rhabdomyosarcoma | 1 | 0.9 |
| **Surgery code (flap)** | Radial forearm flap | 98 | 85.2 |
|  | Anterolateral thigh flap | 7 | 6.1 |
|  | Combined | 5 | 4.3 |
|  | Latissimus dorsi flap | 4 | 3.5 |
|  | Transverse rectus abdominis flap | 1 | 0.9 |
| **Primary site** | Tongue | 28 | 24.3 |
|  | Tonsil | 22 | 19.1 |
|  | Hypopharynx | 25 | 21.7 |
|  | Floor of mouth | 8 | 7.0 |
|  | Palate | 5 | 4.3 |
|  | Maxilla | 4 | 3.5 |
|  | Oropharynx | 4 | 3.5 |
|  | Glottis | 3 | 2.6 |
|  | Buccal | 2 | 1.7 |
|  | Pharynx | 2 | 1.7 |
|  | Pyriform sinus | 2 | 1.7 |
|  | Esophagus | 1 | 0.9 |
|  | Gingiva | 1 | 0.9 |
|  | Larynx | 1 | 0.9 |
|  | Orbit | 1 | 0.9 |
|  | Parotid | 1 | 0.9 |
|  | Scalp | 1 | 0.9 |
|  | Supraglottis | 1 | 0.9 |
|  | Tongue and mouth floor | 1 | 0.9 |
|  | Thyroid | 1 | 0.9 |
|  | Trachea | 1 | 0.9 |
| **Stage (AJCC code)** | 1 | 10 | 8.7 |
|  | 2 | 18 | 15.7 |
|  | 3 | 29 | 25.2 |
|  | 4 | 1 | 0.9 |
|  | 4a | 46 | 40.0 |
|  | 4b | 10 | 8.7 |
|  | 4c | 1 | 0.9 |

Table S2. Distributions of complications

| **Complication type** | **n** | **% of cohort (N = 115)** | **% among complications (N = 30)** |
| --- | --- | --- | --- |
| Leakage / Fistula | 20 | 17.40% | 66.70% |
| Total flap loss | 6 | 5.20% | 20.00% |
| Revision operation | 4 | 3.50% | 13.30% |
| Microvascular compromise | 3 | 2.60% | 10.00% |
| Hematoma | 3 | 2.60% | 10.00% |
| Plate exposure | 1 | 0.90% | 3.30% |
| Scar / arthrosis / stricture | 4 | 3.50% | 13.30% |
| Innominate artery rupture | 1 | 0.90% | 3.30% |
| **Total** | **30** | **26.10%** |  |

Table S3. Internal validation of adjusted models and comparison with the main result(B=1000)

| Endpoint | Model | Apparent AUC | Mean Optimism | Optimism‑Corrected AUC | Calculated AUC (no adjustments) | Δ (Absolute difference) |
| --- | --- | --- | --- | --- | --- | --- |
| Overall Complications | Adjusted for log(SII) + Age≥65 + Area + Size>75 | 0.783 | 0.032 | 0.750 | 0.740 | 0.010 |
| Overall Complications | Adjusted for CONUT + Age≥65 + Area + Size>75 | 0.710 | 0.044 | 0.665 | 0.685 | 0.020 |
| Leakage | Adjusted for log(SII) + Age≥65 + Area + Size>75 | 0.739 | 0.053 | 0.686 | 0.780 | 0.094 |
| Leakage | Adjusted for CONUT + Age≥65 + Area + Size>75 | 0.708 | 0.064 | 0.643 | 0.688 | 0.045 |

Table S4. DeLong test result for comparison of performance between log(SII) and CONUT

| Endpoint | AUC (log(SII), 95% CI) | AUC (CONUT, 95% CI) | Δ(AUC) (95% CI) | Z | p |
| --- | --- | --- | --- | --- | --- |
| Overall Complications | 0.780 (0.686–0.875) | 0.688 (0.569–0.806) | +0.092 (−0.014–0.161) | 1.64 | 0.101 |
| Leakage | 0.740 (0.619–0.861) | 0.685 (0.552–0.817) | +0.055 (−0.068–0.131) | 0.61 | 0.541 |

Table S5. DeLong comparisons of log(SII) vs NLR/PNI. Positive Δ(AUC) favors the first-listed index (log(SII) or CONUT). *Significant difference (p < 0.05)

| Endpoint | Comparison | AUC (Primary) | AUC (Comparator) | Δ(AUC) (95 % CI) | Z | p |
| --- | --- | --- | --- | --- | --- | --- |
| Surgical Complications | log-SII vs NLR | 0.780 | 0.775 | +0.006 (−0.042 – +0.053) | 0.24 | 0.81 |
|  | log-SII vs PNI | 0.780 | 0.681 | +0.461 (+0.276 – +0.646) | 4.89 | < 0.001* |
|  | CONUT vs NLR | 0.688 | 0.775 | −0.087 (−0.177 – +0.003) | −1.89 | 0.059 |
|  | CONUT vs PNI | 0.688 | 0.319 | +0.368 (+0.150 – +0.586) | 3.31 | 0.001* |
| Leakage | log-SII vs NLR | 0.740 | 0.747 | −0.007 (−0.057 – +0.042) | −0.29 | 0.77 |
|  | log-SII vs PNI | 0.740 | 0.690 | +0.430 (+0.209 – +0.651) | 3.82 | < 0.001* |
|  | CONUT vs NLR | 0.685 | 0.747 | −0.063 (−0.168 – +0.043) | −1.17 | 0.244 |
|  | CONUT vs PNI | 0.685 | 0.310 | +0.375 (+0.138 – +0.612) | 3.10 | 0.002* |

Table S6. Temporal stratification by era

| Endpoint | Period | n | Number of events | AUC_log(SII) | AUC_CONUT | CI_log(SII) | CI_CONUT |
| --- | --- | --- | --- | --- | --- | --- | --- |
| Overall complications | Early | 26 | 5 | 0.848 | 0.519 | 0.655 - 1.000 | 0.188 - .850 |
| Leakage | Early | 26 | 3 | 0.768 | 0.688 | 0.450 - 1.000 | 0.318 - 1.000 |
| Overall complications | Late | 89 | 25 | 0.761 | 0.714 | 0.649 - 0.872 | 0.590 - 0.839 |
| Leakage | Late | 89 | 17 | 0.728 | 0.673 | 0.592 - 0.864 | 0.526 - 0.820 |

Table S7. Exploratory binary associations between high inflammatory–nutritional indices and specific postoperative complications. *Significant difference (p < 0.05)

| **Outcome** | **Marker** | **N (event / total, %)** | **OR (95 % CI)** | **p** |
| --- | --- | --- | --- | --- |
| **Flap loss** | High SII (≥ 750) | 6 / 41 (14.6 %) vs 0 / 74 (0 %) | 9.94 (1.06–484.6) | 0.021 * |
|  | High CONUT (≥ 4) | 6 / 27 (22.2 %) vs 0 / 88 (0 %) | 3.49 (0.44–27.8) | 0.14 |
| **Revision surgery** | High SII (≥ 750) | 14 / 41 (34.1 %) vs 0 / 74 (0 %) | 3.83 (1.05–15.8) | 0.034 * |
|  | High CONUT (≥ 4) | 14 / 27 (51.9 %) vs 0 / 88 (0 %) | 2.83 (0.73–10.5) | 0.09 |
| **Microvascular compromise** | High SII (≥ 750) | 4 / 41 (9.8 %) vs 0 / 74 (0 %) | ∞ (1.24–∞) | 0.015 * |
|  | High CONUT (≥ 4) | 4 / 27 (14.8 %) vs 0 / 88 (0 %) | 10.6 (0.81–575.1) | 0.040 * |
| **Hematoma** | High SII (≥ 750) | 3 / 41 (7.3 %) vs 0 / 74 (0 %) | ∞ (0.76–∞) | 0.043 * |
|  | High CONUT (≥ 4) | 3 / 27 (11.1 %) vs 0 / 88 (0 %) | 6.81 (0.34–414.0) | 0.14 |
