# Supplementary figures and images for: Validation of the Controlling Nutritional Status (CONUT) Score and the Systemic Immune-Inflammation Index (SII) for Predicting Leakage and Surgical Complications After Head and Neck Free Flap Reconstruction: A Pilot Study
Source: Medicina (Kaunas). 2025 Nov 22;61(12):2084. doi: 10.3390/medicina61122084 (PMC12734734; doi:10.3390/medicina61122084)

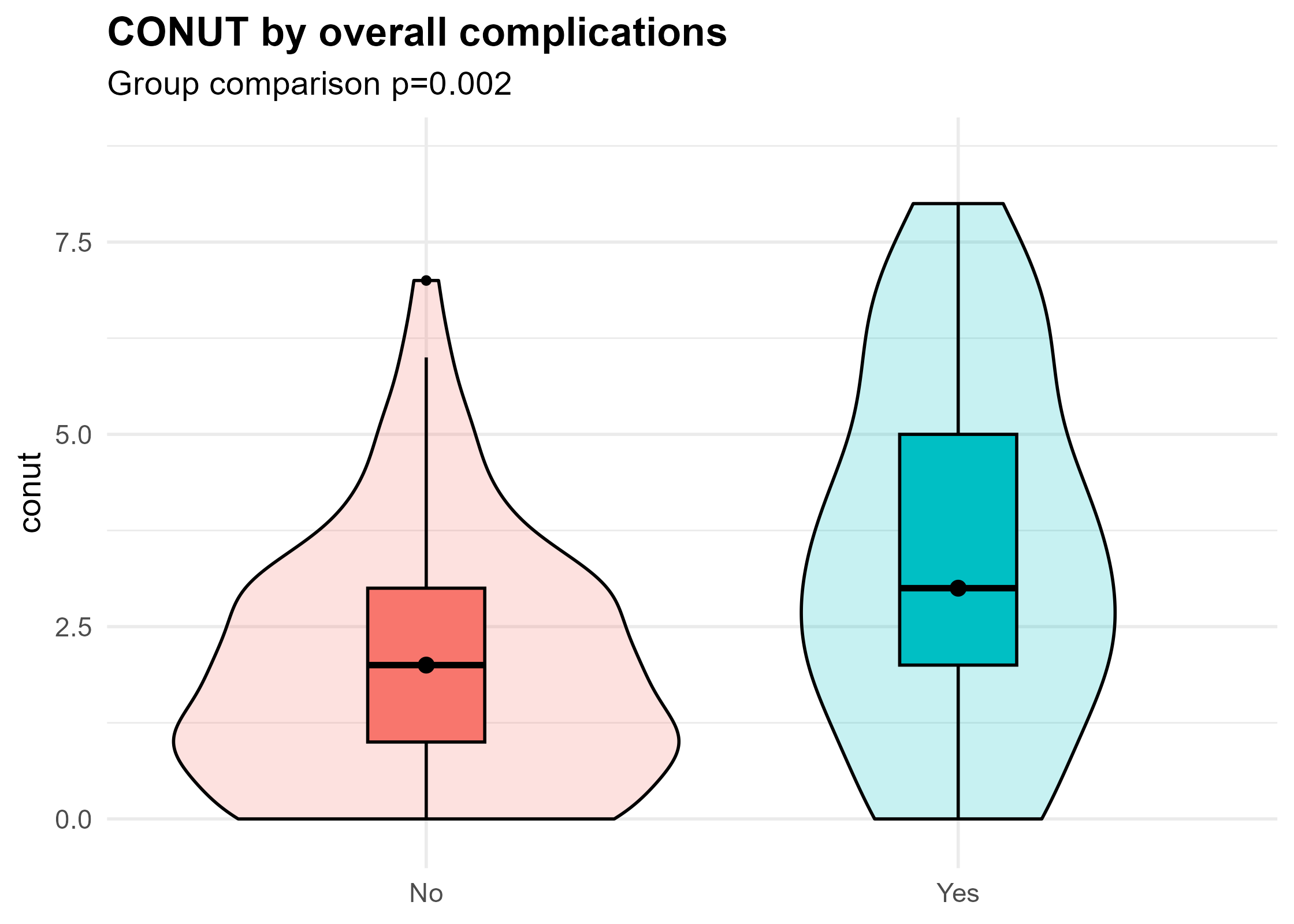

Supplement: Supplementary file 1 [file medicina-61-02084-s001.zip › final medicina-3953298-supplementary/FigS1A_CONUT_vs_Complications.png]

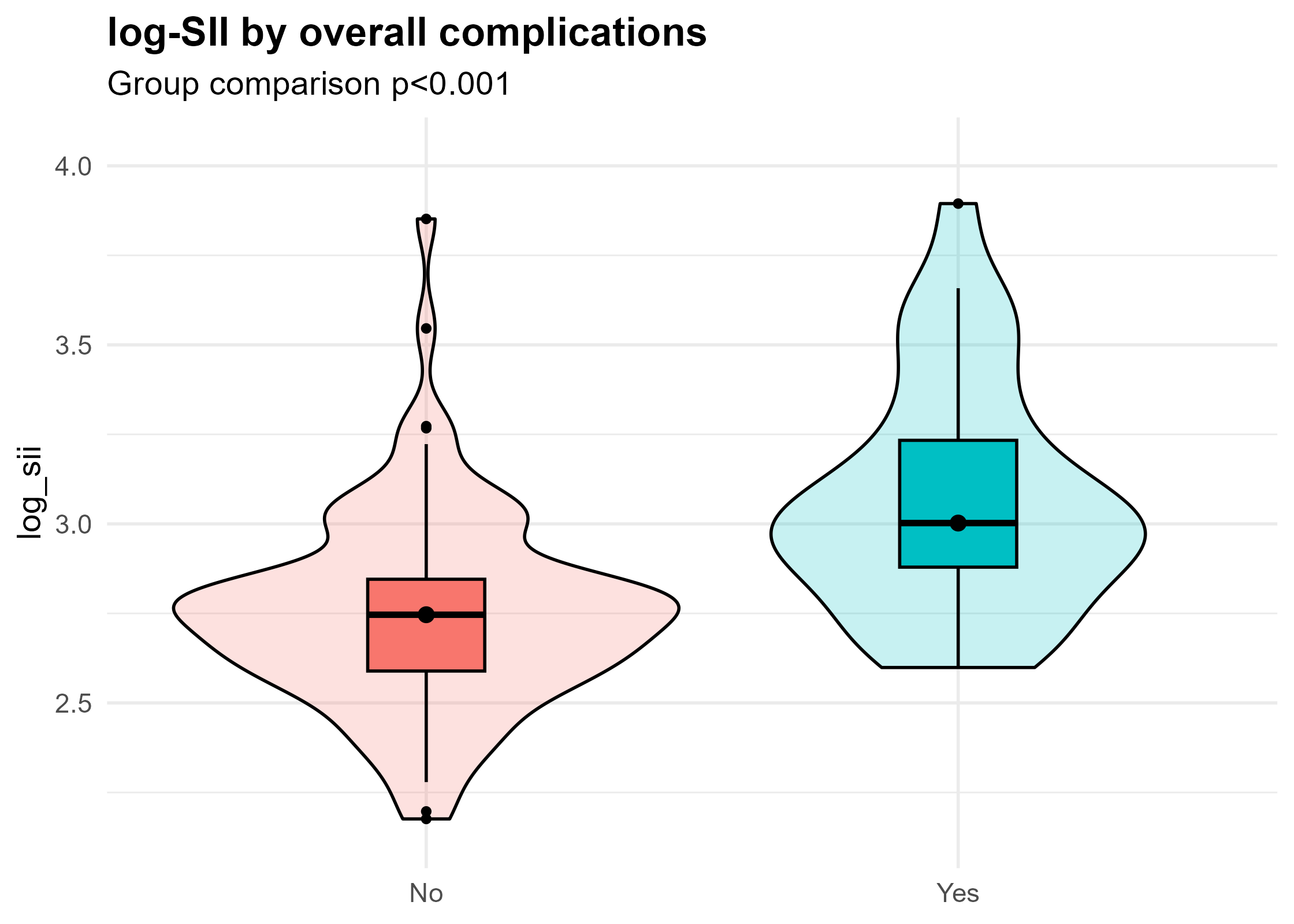

Supplement: Supplementary file 1 [file medicina-61-02084-s001.zip › final medicina-3953298-supplementary/FigS1B_logSII_vs_Complications.png]

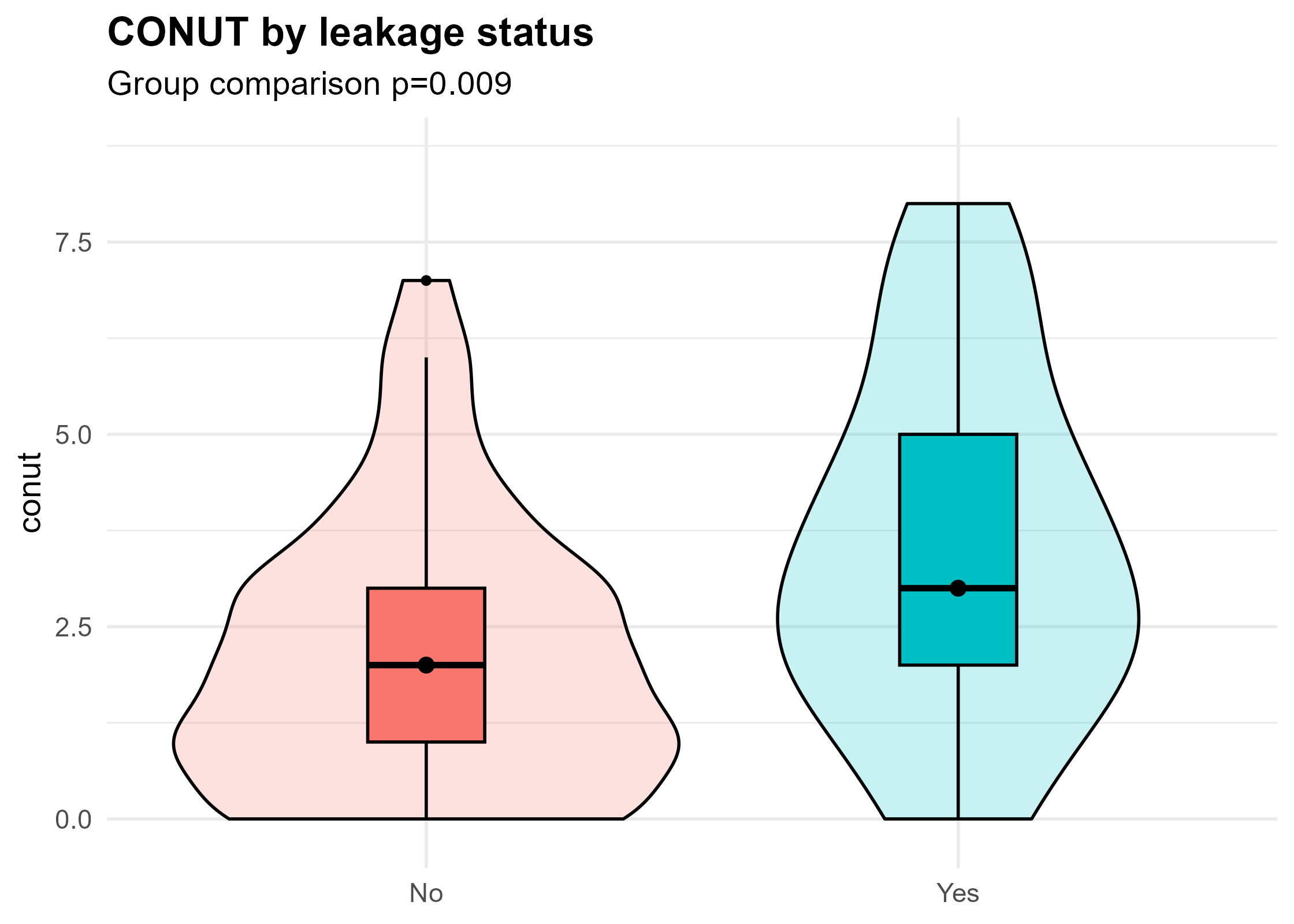

Supplement: Supplementary file 1 [file medicina-61-02084-s001.zip › final medicina-3953298-supplementary/FigS1C_CONUT_vs_Leakage.png]

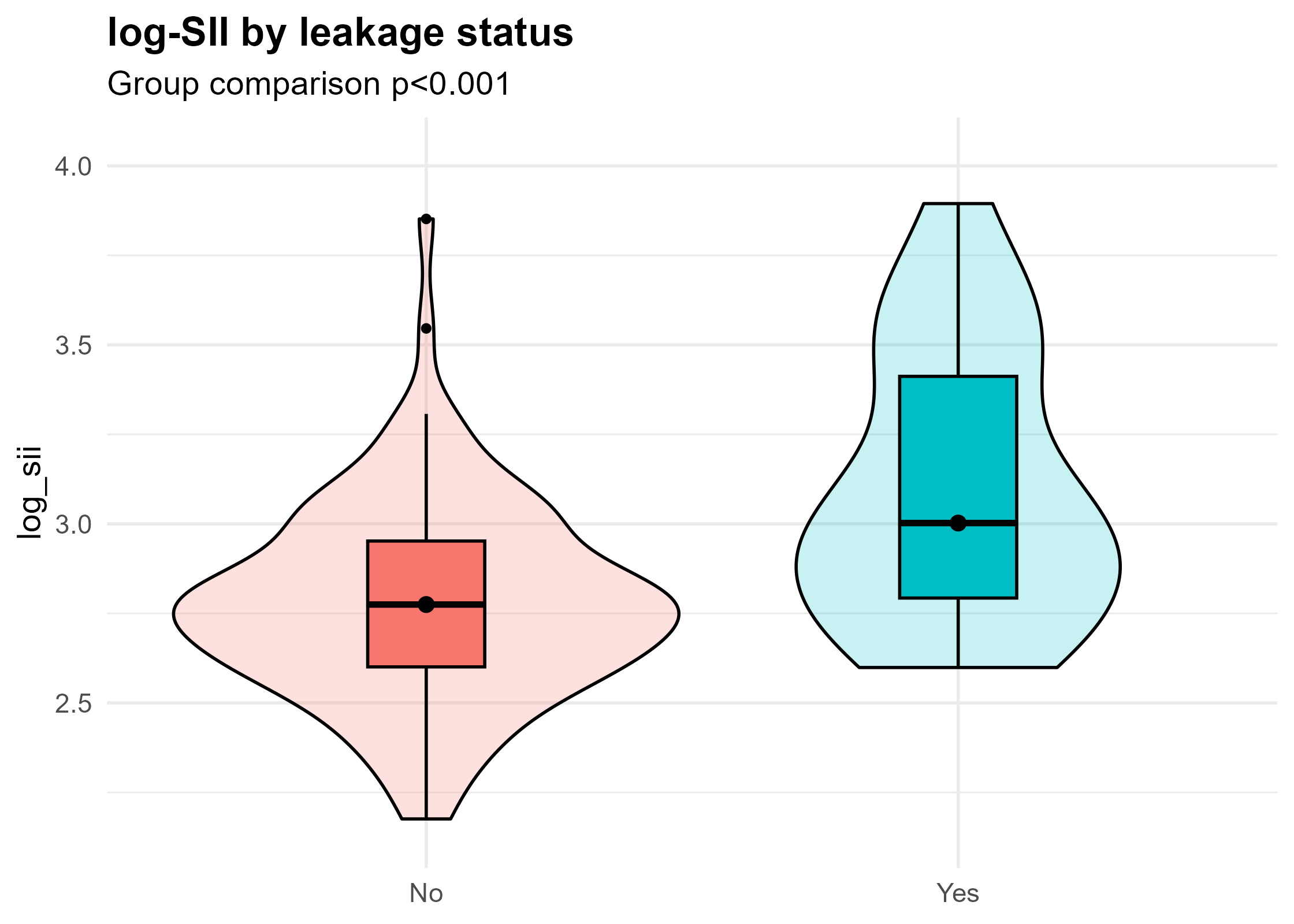

Supplement: Supplementary file 1 [file medicina-61-02084-s001.zip › final medicina-3953298-supplementary/FigS1D_logSII_vs_Leakage.png]

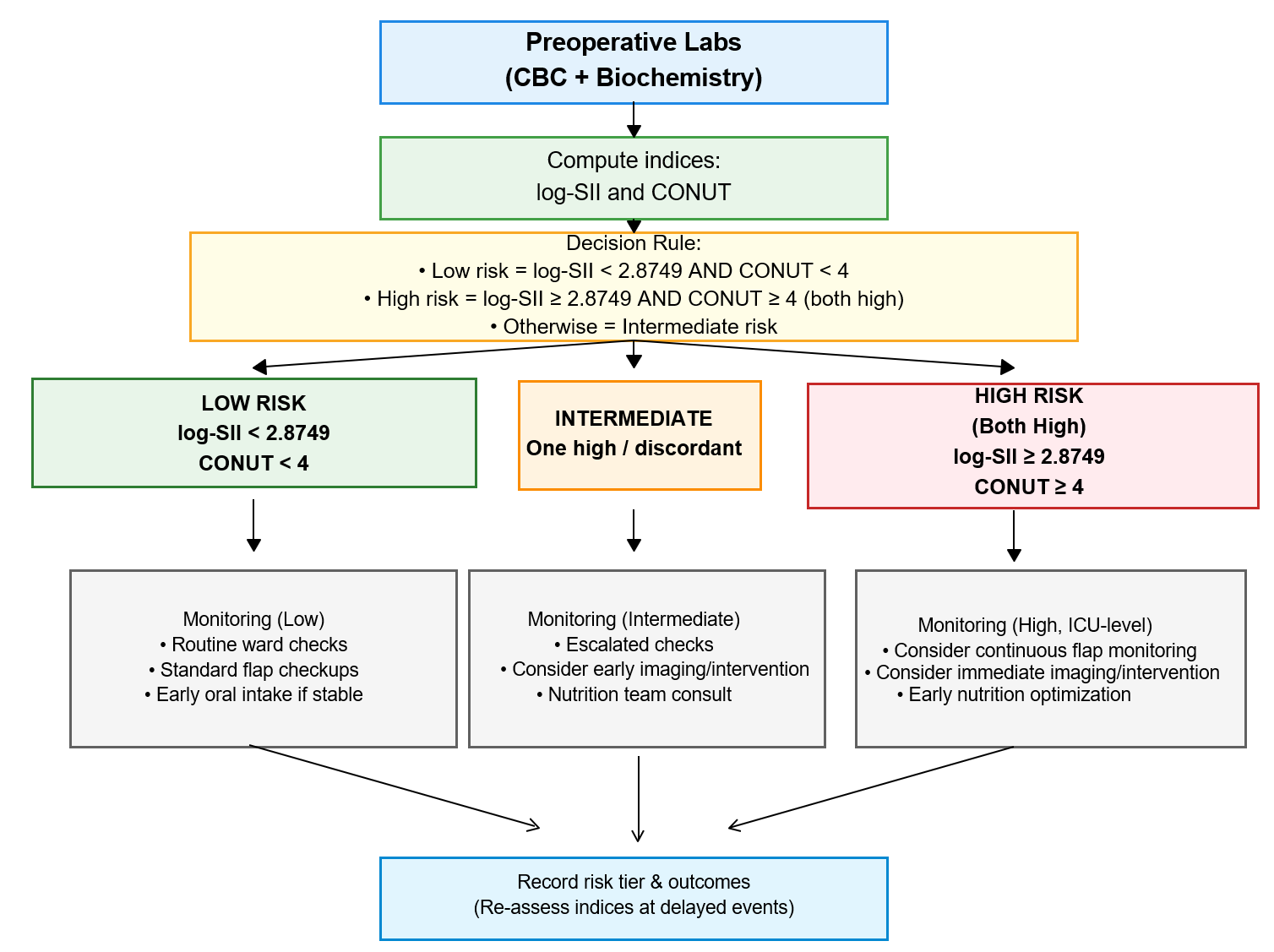

Supplement: Supplementary file 1 [file medicina-61-02084-s001.zip › final medicina-3953298-supplementary/figureS2.tiff]
